# Simultaneous Osteosynthesis and Distal Femoral Osteotomy in a Patient With Distal Femoral Fracture Combined With Valgus Knee Deformity: A Case Report

**DOI:** 10.7759/cureus.65006

**Published:** 2024-07-20

**Authors:** Kentaro Fujita, Kenji Fujita, Daiki Kamata, Hidetoshi Yasutake, Satoru Demura

**Affiliations:** 1 Department of Orthopaedic Surgery, Ishikawa Prefectural Central Hospital, Kanazawa, JPN; 2 Department of Orthopaedic Surgery, Graduate School of Medical Sciences, Kanazawa University, Kanazawa, JPN

**Keywords:** osteosynthesis, distal femoral osteotomy, osteoarthritis of the knee, medial closing-wedge distal femoral osteotomy, distal femoral fracture

## Abstract

Distal femoral fractures are commonly treated with osteosynthesis. However, in older patients with osteoarthritis of the knee, acute primary total knee arthroplasty (TKA) may be performed to treat these fractures. Notably, no studies have documented the use of osteosynthesis in combination with distal femoral osteotomy (DFO) for treating distal femoral fractures in patients with knee osteoarthritis. This report presents the case of a 66-year-old woman with lateral compartment osteoarthritis accompanied by severe valgus knee deformity who underwent osteosynthesis for a distal femoral fracture combined with medial closing-wedge distal femoral osteotomy (MCWDFO) to correct the knee valgus deformity. She experienced a distal femoral fracture (AO/OTA 33B1.1) of the right knee because of a fall. Before the injury, she exhibited a limp due to severe knee pain with some limitations in knee flexion. Non-weight-bearing radiographs of the entire lower extremity suggested a percentage mechanical axis (%MA) of 115%, indicating severe valgus deformity. On day nine after the injury, we performed osteosynthesis for the distal femoral fracture and conducted an MCWDFO to correct the right knee valgus deformity. After MCWDFO, the %MA was corrected to 70%. Partial weight-bearing was initiated three weeks postoperatively and progressed to full weight-bearing at six weeks. To facilitate bone healing, low-intensity pulsed ultrasound (LIPUS) was applied for three months after surgery. Bone union was successfully achieved by month five. Some medial knee pain persisted for six months after surgery; nonetheless, the patient could walk without a limp. We considered that the integration of MCWDFO with osteosynthesis could provide a treatment option for patients with distal femoral fractures and lateral compartment osteoarthritis.

## Introduction

Distal femoral fractures represent approximately 3% to 6% of all femoral fractures. They are typically caused by high-energy trauma, such as traffic accidents, or low-energy trauma, such as falls, in patients with osteoporosis [1]. Surgical treatment for distal femoral fractures generates better outcomes than conservative treatment, emphasizing the importance of anatomically reducing the joint surface and restoring the leg length, rotation, and alignment [2,3].

Medial closing-wedge distal femoral osteotomy (MCWDFO) is conducted in patients with valgus deformities caused by lateral compartment osteoarthritis of the knee. Traditionally, the use of MCWDFO has been limited owing to the difficulty of accurate correction, the risk of postoperative correction loss, and complications such as nonunion [4,5]. However, advancements such as specialized locking plates have enabled more stable results [6].

In this report, we present the case of a distal femoral fracture combined with lateral knee osteoarthritis and severe knee valgus deformity. We performed a one-stage MCWDFO to correct the knee valgus deformity, in addition to osteosynthesis for the distal femoral fracture. The procedure resulted in successful outcomes, which are discussed in detail with reference to the existing literature.

## Case presentation

**Figure 1:**
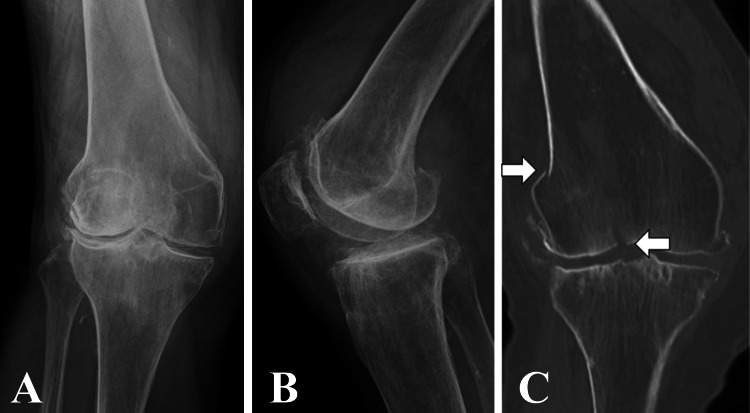
Preoperative images Preoperative anteroposterior (A) and lateral (B) radiographs, and anteroposterior CT scan (C) indicates distal femoral fracture of AO/OTA type 33B1.1. The right knee exhibits severe lateral compartment osteoarthritis (Kellgren–Lawrence grade 4). CT, computed tomography

A 66-year-old woman had a fall and presented to our hospital with severe right knee pain. She experienced difficulty in walking. She was 162.0 cm tall and weighed 71.0 kg; thus, her body mass index was 27.05 kg/m^2^. In her childhood, she had sprained her right knee joint; this led to the development of a valgus deformity of the knee. Before the injury, she exhibited a limp due to severe knee pain with some limitations in knee flexion. The initial plain radiographs and computed tomography (CT) suggested a distal femoral fracture of AO/OTA type 33B1.1 (Figure 1).

**Figure 2:**
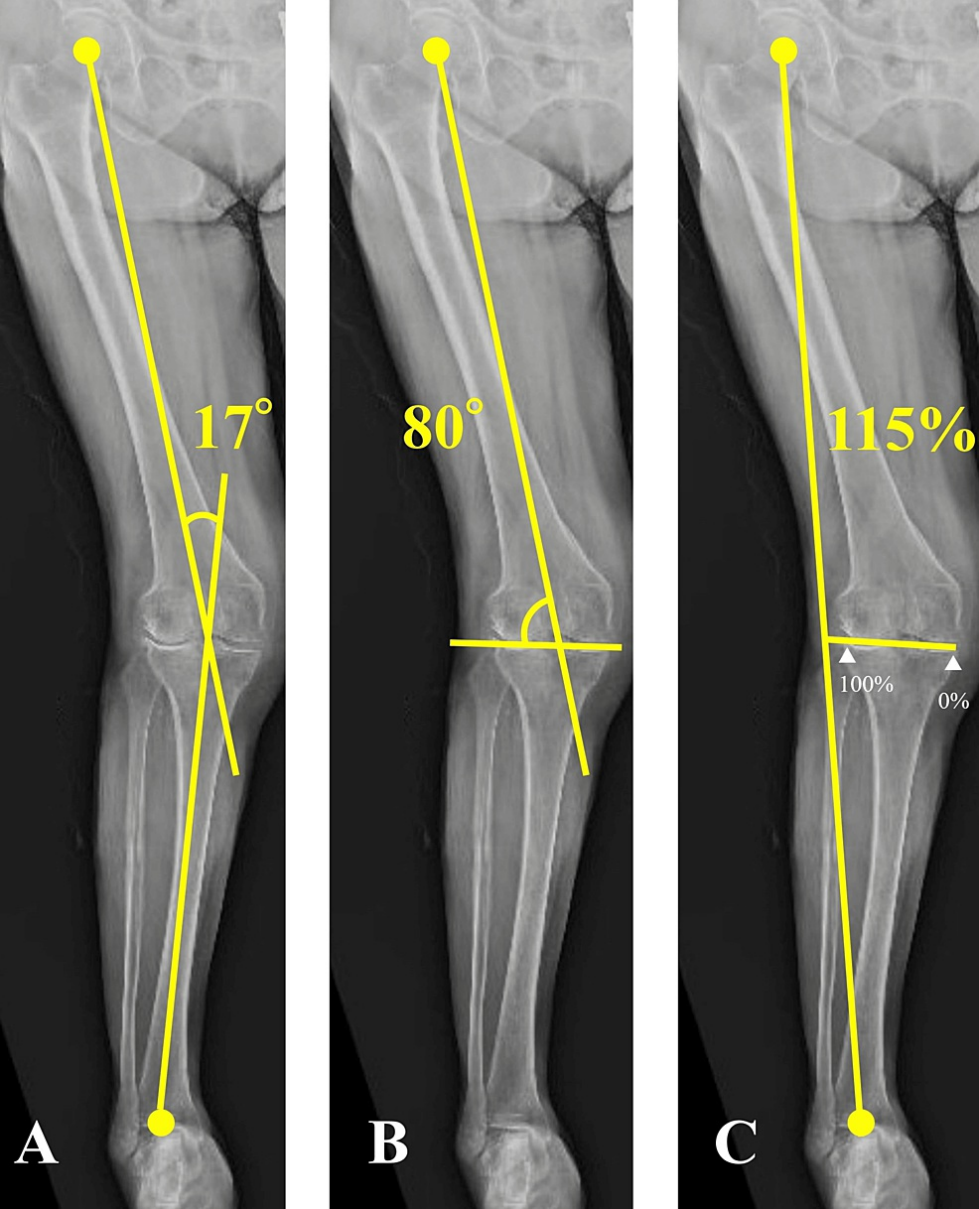
Preoperative non-weight-bearing radiograph of the entire lower extremity (A) HKA of 17°; (B) mLDFA of 80°; (C) %MA of 115%. HKA, hip-knee-ankle angle; mLDFA, mechanical lateral distal femoral angle; %MA, percentage mechanical axis

**Figure 3:**
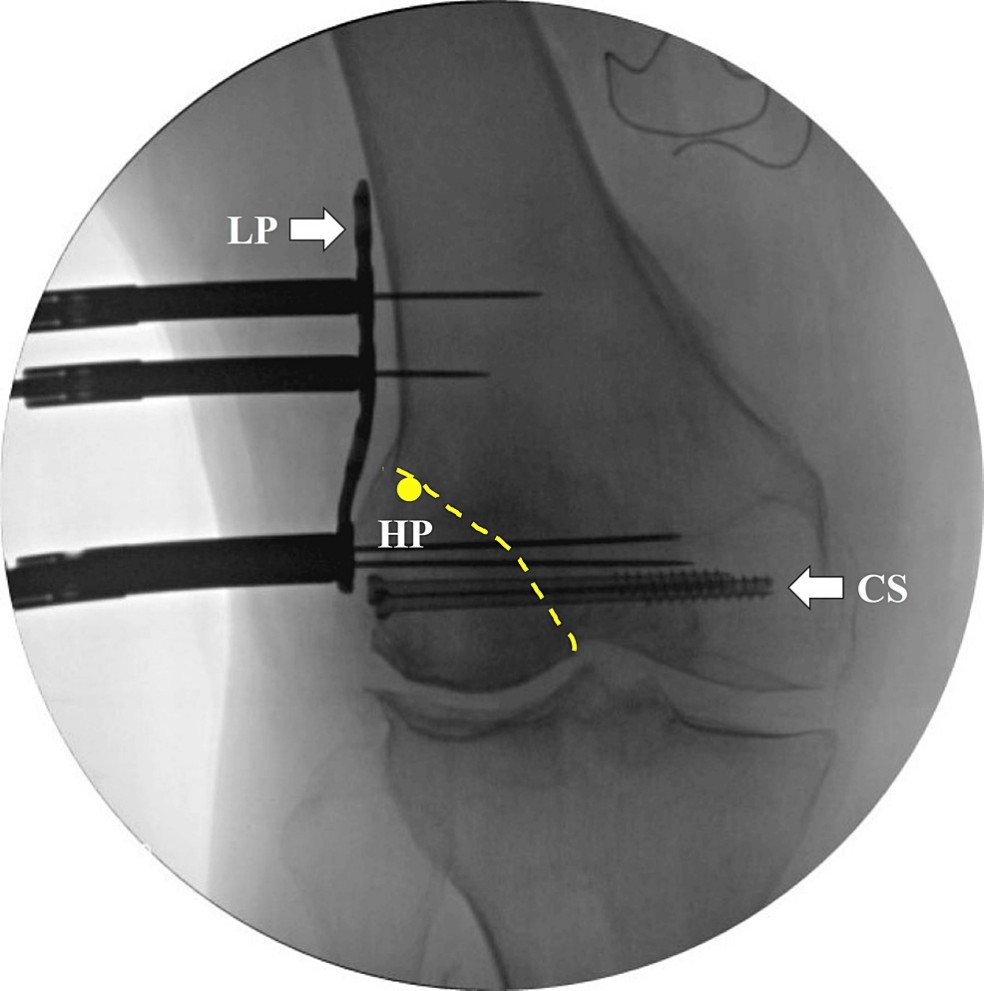
Intraoperative fluoroscopic image The dotted yellow line indicates the fracture line. The optimal hinge point is slightly below the top edge of the lateral condyle and 5 mm inward from the lateral cortex, which is located on the fracture line in this case. Two partially threaded 5.0 mm cannulated screws were placed into the bone fragments at the articular surface to achieve interfragmentary compression. The lateral plate was temporarily secured using 1.4 mm K-wires. HP, hinge point; LP, lateral plate; CS, cannulated screws; K-wire, Kirschner wires

Her right knee exhibited severe lateral compartment osteoarthritis (Kellgren-Lawrence grade 4) on plain radiography (Figure 1). Further imaging with non-weight-bearing radiography of the entire lower extremity suggested a hip-knee-ankle angle (HKA) of 17° (Figure 2A), mechanical lateral distal femoral angle (mLDFA) of 80° (Figure 2B), and percentage mechanical axis (%MA) of 115% (Figure 2C), indicating severe valgus deformity. On day nine after the injury, we performed osteosynthesis for the distal femoral fracture and conducted an MCWDFO to correct the right knee valgus deformity. In preoperative planning, the correction targets were set at an mLDFA of 94° and %MA of 70%, requiring 14° correction. The patient underwent surgery in the supine position under general anesthesia, without the use of a tourniquet. To achieve interfragmentary compression, we inserted two 5.0 mm partially threaded cannulated screws (Asnis III: Stryker, Kalamazoo, MI, USA) into the bone fragments at the articular surface. Subsequently, we positioned a lateral plate (Tris small plate; Olympus Terumo Biomaterials, Tokyo, Japan) designed for MCWDFO hinge fractures and temporarily secured it with 1.4 mm Kirschner wires (K-wire; Figure 3).

**Figure 4:**
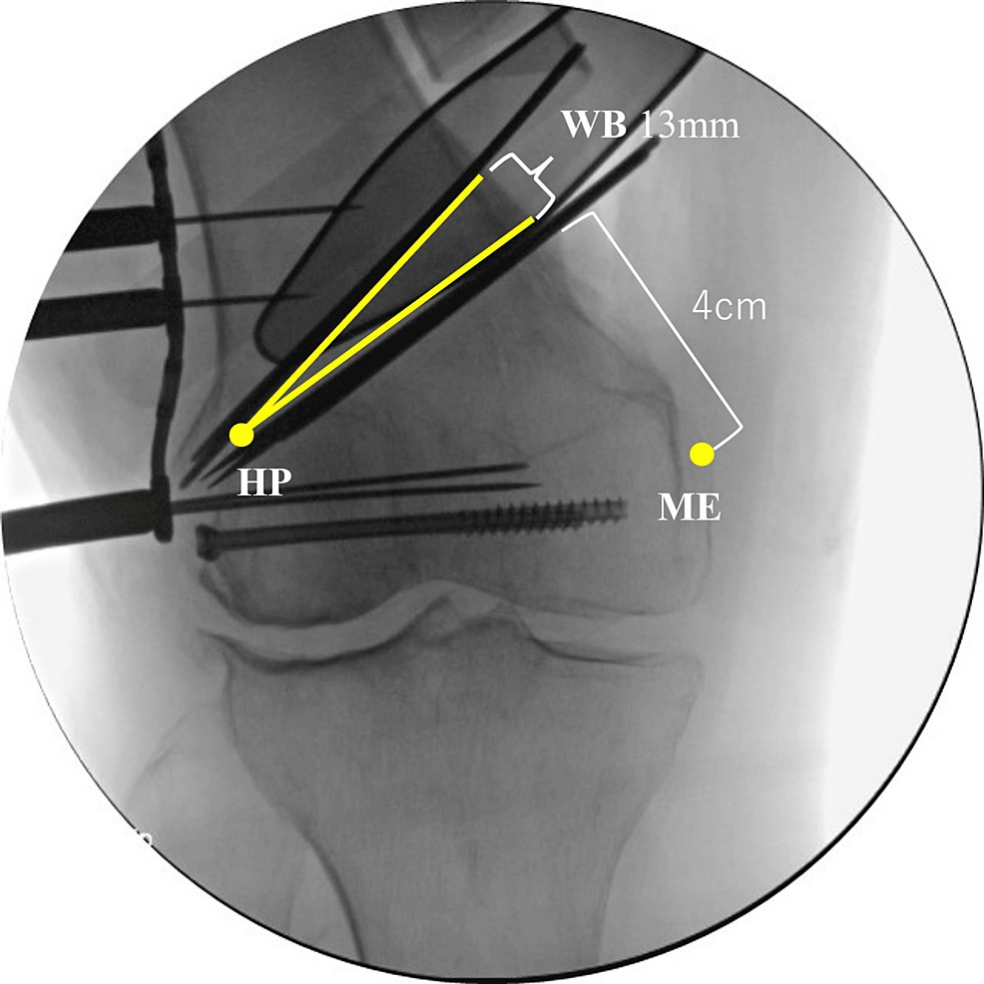
Intraoperative fluoroscopic images Two K-wires were inserted 4 cm above the medial femoral epicondyle and oriented toward the lateral hinge point. The yellow line indicates the osteotomy line. We conducted a single-plane osteotomy, followed by careful closure of the wedge osteotomy site. HP, hinge point; WB, wedge base; ME, medial femoral epicondyle

MCWDFO was conducted using a 10 cm anteromedial longitudinal skin incision. We positioned a lateral hinge point slightly below the top edge of the lateral condyle and 5 mm inward from the lateral cortex. We inserted two K-wires 4 cm above the medial femoral epicondyle and oriented them toward the lateral hinge point. Furthermore, two other K-wires were placed proximal to the distal K-wire with the aid of a wedge-cutting guide. A 13 mm wedge base was located at the medial cortex. Single-plane osteotomy was conducted, followed by careful closure of the wedge osteotomy site (Figure 4).

**Figure 5:**
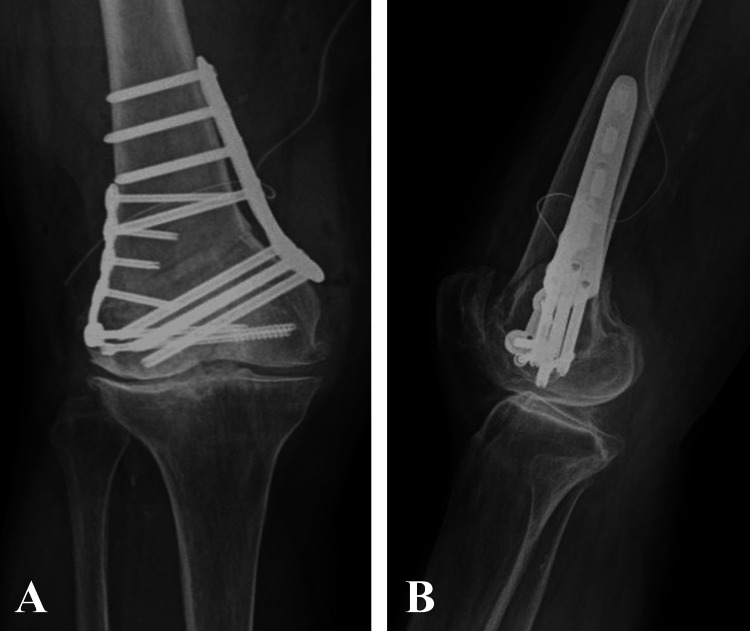
Postoperative anteroposterior (A) and lateral (B) radiographs

We achieved fixation using the Tris-Medial DFO plate system (Olympus Terumo Biomaterials, Tokyo, Japan) - an anatomical locking plate specifically designed for DFO. Finally, the lateral plate was bent, and locking screws were inserted (Figure 5).

**Figure 6:**
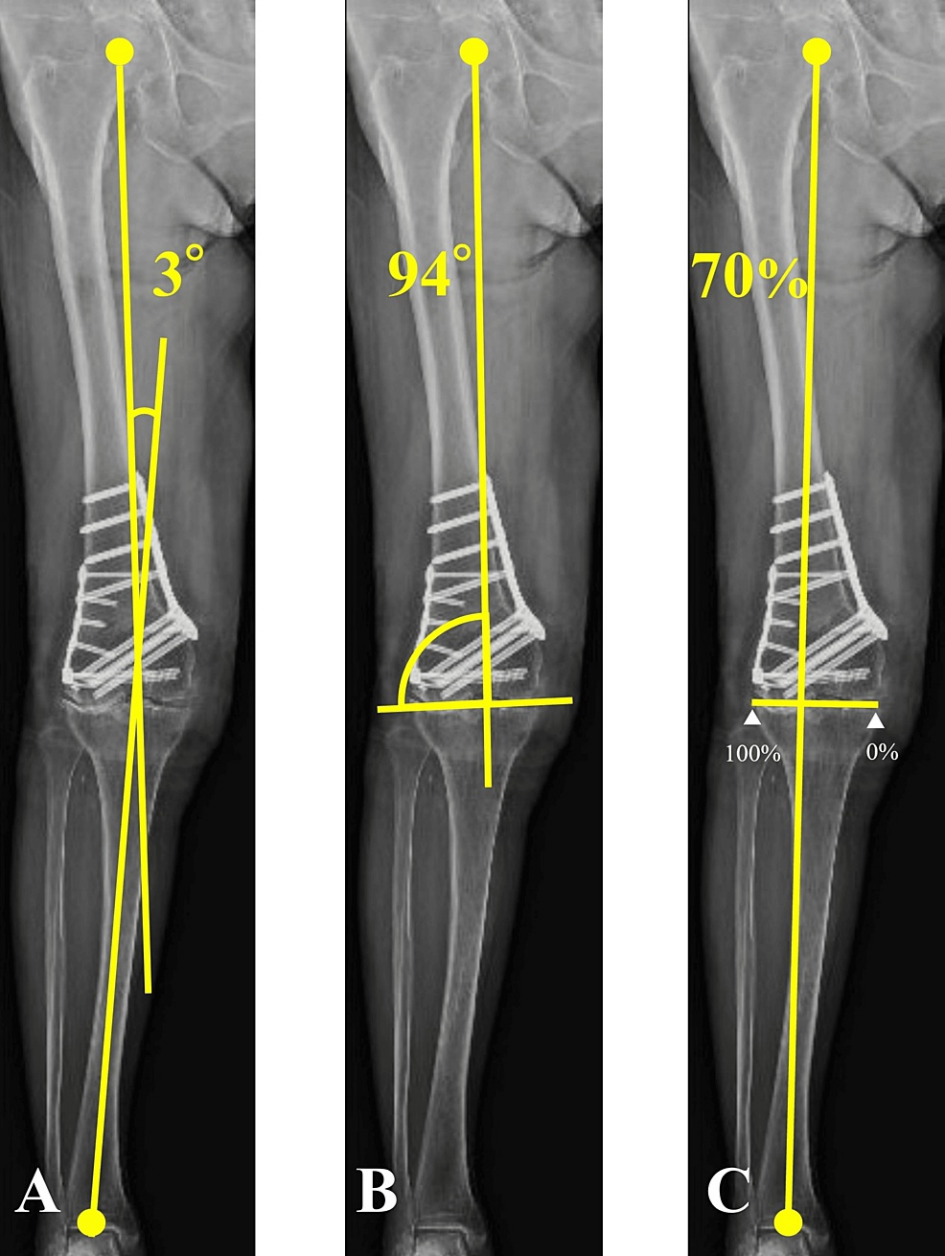
Postoperative weight-bearing radiograph of the entire lower extremity Varus correction is achieved. The yellow lines represent the mechanical axis. (A) HKA of 3°; (B) mLDFA of 94°; (C) %MA of 70%. HKA, hip-knee-ankle angle; mLDFA, mechanical lateral distal femoral angle; %MA, percentage mechanical axis

The patient began range-of-motion exercises on the first-day post-surgery, gradually increasing the intensity based on tolerance. Partial weight-bearing was allowed after three weeks post-surgery, with full weight-bearing allowed after six weeks. After MCWDFO, the HKA, mLDFA, and %MA were corrected to 3°, 94°, and 70%, respectively, verified by full-length, weight-bearing radiography (Figure 6).

Eight weeks post-surgery, the patient was discharged home and continued with outpatient rehabilitation at the hospital. Low-intensity pulsed ultrasound (LIPUS) therapy (Accellus; Nippon Sigmax, Osaka, Japan) was administered for three months after surgery to promote bone healing, which was confirmed at five months. Six months post-surgery, the patient demonstrated a knee flexion of 135° and extension of −5°. The knee injury and osteoarthritis outcome scores (KOOS) were as follows: symptoms, 71.4; pain, 69.4; function in daily living, 82.4; function in sport and recreation, 70.0; and knee-related quality of life, 75.0. The patient experienced mild pain in the medial compartment but could walk without a limp.

## Discussion

To the best of our knowledge, this is the first report of one-stage MCWDFO to correct a knee valgus deformity, in addition to osteosynthesis for a distal femoral fracture. The issue in this case was how to manage the osteoarthritis of the knee with severe valgus deformity, which had been identified before the distal femoral fracture occurred.

The surgical management of distal femoral fractures involves osteosynthesis, as anticipated in this case. However, the patient had knee pain and limping caused by severe lateral compartment osteoarthritis. Thus, total knee arthroplasty (TKA) would likely have been necessary in the near future.

Alternatively, acute primary TKA has been reported for distal femoral fractures in older adults with knee osteoarthritis [7]. The advantages of TKA for distal femoral fractures, particularly in older adults, include the ability to bear weight immediately and mobilize early; this preserves knee joint function and alleviates pain from osteoarthritis. However, if acute primary TKA had been performed in this case, the severity of the valgus deformity would have warranted the need for constrained condylar knee (CCK) implants. The major concern regarding the use of CCK implants is long-term loosening. The survival rates of CCK implants free of reoperation are 90.0% at 10 years and 72.8% at 20 years, highlighting concerns regarding their long-term durability [8].

Therefore, we planned a one-stage MCWDFO to correct the knee valgus deformity, in addition to osteosynthesis for the distal femoral fracture. MCWDFO can prevent osteoarthritis progression and reduce the likelihood of future TKA [9]. Furthermore, despite the need for TKA, correcting valgus deformity through MCWDFO might obviate the need for CCK implants. The patient had a partial intra-articular fracture extending from the femoral condyle to the lateral epicondyle. Fujita et al. demonstrated that the optimal hinge point for MCWDFO is located at the distal lateral cortex endpoint of the femur, which corresponded to the fracture line in this case [10]. Conducting MCWDFO on this knee is equivalent to a hinge fracture; therefore, we utilized double-plate fixation using a lateral plate for hinge fractures, in addition to inserting the cannulated screws into the articular surface [10]. This approach was expected to provide sufficient fixation strength. Furthermore, LIPUS was used to achieve bone union within five months after surgery. The optimal correction angle has been established for valgus high tibial osteotomy [11]. By contrast, the varus correction angle for the knee remains unclear. However, several researchers have targeted a %MA of 50% in DFO for valgus alignment while treating concomitant lateral compartment knee osteoarthritis. In this case, only femoral correction was planned. Thus, we targeted an mLDFA at the upper normal limit of 92°, considering osteoarthritis progression in the medial compartment because of the joint line obliquity angle [12]. Comparing the range of motion and clinical outcomes of the knee before injury was not feasible in this case. Six months postoperatively, some medial knee pain was observed, with a marginally lower KOOS pain score of 69.4. This score was attributed to a shift in the mechanical axis of the lower extremity medially, despite improved pain. Valgus deformity correction substantially improved gait, and the KOOS function of daily living score was high (82.4), indicating no impairment in daily activities.

In this case, a one-stage MCWDFO combined with osteosynthesis could be performed because the osteotomy and fracture lines did not intersect. However, this approach is difficult to adopt when fracture lines cross the osteotomy line or in cases of complete articular fractures. In the future, we will continue to investigate candidate cases that can be effectively treated with this approach.

## Conclusions

This case report demonstrates the successful outcome of a one-stage MCWDFO to correct knee valgus deformity combined with osteosynthesis for a distal femoral fracture in a patient with severe lateral knee osteoarthritis. The procedure significantly improved the patient’s pain, knee function, and walking ability, potentially reducing the need for future TKA. Further research is needed to assess the efficacy and safety of this approach.
